# Primary treatment of pelvic organ prolapse: pessary use versus prolapse surgery

**DOI:** 10.1007/s00192-017-3372-x

**Published:** 2017-06-09

**Authors:** Anne-Lotte W. M. Coolen, Stephanie Troost, Ben Willem J. Mol, Jan- Paul W. R. Roovers, Marlies Y. Bongers

**Affiliations:** 1 Department of Gynecology and Obstetics, Máxima Medical Centre, De Run 4600, 5500 MB Veldhoven, The Netherlands; 2 Department of Gynaecology and Obstetrics, The Robinson Research Institute, School of Paediatrics and Reproductive Health, University of Adelaide, Adelaide, SA 5000 Australia; 3 Department of Gynaecology and Obstetrics, Academic Medical Centre Amsterdam, Meibergdreef 9, 1105 AZ Amsterdam, The Netherlands; 4 Department of Gynaecology and Obstetrics, Grow School of Oncology and Biological Diversity, Maastricht University, Minderbroedersberg 4-6, 6211 LK Maastricht, The Netherlands

**Keywords:** Pelvic organ prolapse, Pessary, Prolapse surgery

## Abstract

**Introduction and hypothesis:**

The objective of this study was to compare the functional outcomes after pessary treatment and after prolapse surgery as primary treatments for pelvic organ prolapse (POP).

**Methods:**

This was a prospective cohort study performed in a Dutch teaching hospital in women with symptomatic POP of stage II or higher requiring treatment. Patients were treated according to their preference with a pessary or prolapse surgery. The primary endpoint was disease-specific quality of life at 12 months follow-up according to the prolapse domain of the Urogenital Distress Inventory (UDI) questionnaire. Secondary outcomes included adverse events and additional interventions. To show a difference of ten points in the primary outcome, we needed to randomize 80 women (power 80%, α 0.05, taking 10% attrition into account).

**Results:**

We included 113 women (74 in the pessary group, 39 in the surgery group). After 12 months, the median prolapse domain score was 0 (10th to 90th percentile 0–33) in the pessary group and 0 (10th to 90th percentile 0–0) in the surgery group (*p* < 0.01). Differences in other domain scores were not statistically significant. In the pessary group, 28% (21/74) of the women had a surgical intervention versus 3% (1/39) reoperations in the surgery group (*p* = 0.01).

**Conclusions:**

In women with POP of stage II or higher undergoing surgery, prolapse symptoms were less severe than in those who were treated with a pessary, but 72% of women who were treated with a pessary did not opt for surgery.

*Trial registration number:* Dutch trial register NTR2856.

## Introduction

Pelvic organ prolapse (POP) is a common condition. In a general Dutch female population aged 45–85 years, 75% of women had some degree of POP [1, 2]. Around 10% of women undergo surgery at some time in their lives for the management of prolapse or urinary incontinence [2]. Pessaries have been used as conservative treatment since the beginning of recorded history. Pessaries are used for the treatment of POP by more than 85% of gynaecologists and 98% of urogynaecologists [3, 4]. Patient preference plays a very important role in the willingness to try a pessary according to a Dutch study showing that 48% of treatment-naive women prefer surgery, 36% prefer pessary treatment and 16% have no preference [5]. However, relevant cost savings can be realized as pessary therapy is inexpensive and the costs are mainly related to doctor visits and the treatment of side effects, which would be even lower in self-managing patients (van de Waarsenburg and van de Vaart, unpublished, 2014. Pessary or surgery for symptomatic organ prolapse: study protocol, version 1.4). Although pessaries have been reported to be effective in reducing prolapse symptoms [6–11], 20–50% of women discontinue pessary use within 1 year [12, 13]. Side effects have been reported to occur in half of the women and are the main reason for discontinuation [12].

The aim of POP surgery is to reduce bother related to pelvic floor symptoms by restoring the anatomy of the vagina and surrounding visceral organs. Unfortunately, POP surgery can be associated with complications. Furthermore, there are significant cost implications for prolapse surgery, particularly when the index surgery has a quoted failure rate of up to 30% [11]. According to two prospective trials comparing pessary treatment with POP surgery, women treated with pessary and women treated with surgery report similar improvements in urinary and bowel symptoms, sexual function, and quality of life [11, 14]. There are no randomized controlled trials (RCT) comparing pessary treatment and surgery. Although POP surgery has several advantages over pessary treatment, the risk of complications is higher and it might be less cost-effective. Since previous studies have shown promising results with pessary treatment, it might be an equivalent option in the treatment of POP, probably with less risk and lower cost.

In view of this dilemma, we decided to start a study with a prospective cohort group treated with either pessary or POP surgery to individualize counselling on treatment options and to guide patients better in the treatment decision process. The aim of this study was to compare disease-specific quality of life after 12 months between pessary treatment and surgery in women treated for POP. In this superiority study, we assumed that POP surgery would lead to a higher disease-specific quality of life than pessary treatment at 12 months after the start of treatment.

## Materials and methods

We performed this RCT comparing pessary treatment and prolapse surgery as primary treatments for POP in a teaching hospital in The Netherlands. The study was approved by the ethics committee and is registered with the Dutch trial register (NTR2856). Women with a treatment preference who did not consent to randomization were asked to follow the same study protocol as part of a prospective cohort group. During the study, many women were found to have a strong preference for one or other of the treatment options, and did not consent to randomization. Therefore, the RCT was prematurely ended, resulting in a prospective cohort group, including 6 randomized women and 107 women treated according to their preference.

Women with symptomatic POP who preferred to undergo treatment were eligible for the trial. Symptomatic POP was defined as Pelvic Organ Prolapse Quantification (POP-Q) stage II or higher with bothersome urogenital symptoms. Women who had undergone previous surgery for correction of POP or urinary incontinence, or who had previously been treated with a pessary were excluded, as were patients with a contraindication to surgical intervention. An isolated rectocele without prolapse of any other compartment was also an exclusion criteria, since if a solitary rectocele is present there may be insufficient support for a pessary [11].

Eligible women with symptomatic POP who met the inclusion criteria were counselled about POP and their condition by their own gynaecologist, one of a team of three urogynaecologists. Then the treatment options expectant management, pessary treatment and surgery were explained. After the consultation, women received written information about their condition and about the trial. They then had time to consider participation in the trial, being randomized or being part of the prospective cohort group. The interventions were either pessary treatment or POP surgery following patient preference. If the patient consented to randomization she signed written informed consent and was randomized. Randomization was performed using opaque sealed envelopes. The treatment allocation ratio was 1:1 to either pessary treatment or surgery. No block randomization was performed. Patients and physicians were not blinded.

### Pessary treatment

According to the judgement of the gynaecologist, either a shelf (Falk) or a ring pessary (with or without central support) could be used. No other pessaries were placed during the trial. Preferably a ring pessary was placed. According to the judgement of the physician, a ring pessary with central support was placed in patients with apical descent, and a shelf (Falk) pessary was placed in patients with apical descent, extensive prolapse or lack of support of the ring pessary. Pessary fitting was performed at the outpatient clinic after fitting. A test pessary was used to determine the correct size of the pessary. The patient tested this pessary while walking around for 30 min. If the right size was found, the pessary was inserted and the fitting was considered successful. The type and size of pessary were recorded, as well as the number of different types and sizes. Side effects during pessary treatment including discharge, pain and blood loss were also recorded. After placement, all patients received instructions about pessary treatment. If the initial fit was unsuccessful another size or the other pessary type could be used, according to the judgement of the gynaecologist. Discontinuation and expulsion were recorded, and if pessary treatment was not successful, the reason for failure was also recorded.

A follow-up visit 6 weeks after placement was planned, and patients were instructed to return to the clinic if they had any complaints or if they lost the pessary. If the patients was satisfied with the pessary, follow-up visits every 3–4 months were planned for pessary cleaning and vaginal inspection. During the follow-up period, the type or size of the pessary could be changed. All participating physicians were skilled in pessary fitting and had performed at least 100 pessary fittings prior to the start of the trial.

### Surgical intervention

The surgical intervention consisted of correction of all compartments that required surgery. The decisions as to which technique to use and which compartments to treat were left to the discretion of the gynaecologist, and depended on the results of the physical examination and the complaints. Cystocele repair involved conventional anterior colporrhaphy. For uterine descent, different techniques were allowed. These techniques could be vaginal hysterectomy with vault suspension or uterus-preserving techniques such as sacrospinous fixation, the Manchester-Fothergill procedure or a laparoscopic sacrohysteropexy. A coexisting rectocele was treated with conventional posterior colporrhaphy. When stress incontinence was diagnosed prior to surgery, the patient and surgeon decided whether to perform a concomitant incontinence procedure or whether first to perform prolapse surgery only with additional incontinence surgery later.

All procedures were performed under general anaesthesia or spinal anaesthesia. Prophylactic antibiotics were given peroperatively (metronidazole/cefalozine). As prophylaxis for thromboembolism, low molecular weight heparin was administered subcutaneously peroperatively and postoperatively. The data collected included the type of surgery, procedure time, estimated blood loss, length of hospital stay and perioperative complications. A urethral catheter was left in situ and was removed on the second postoperative day in women with anterior colporrhaphy or on the first day in other patients. If the procedure was complicated by a bladder lesion, the catheter was removed after 1 week. If urine retention occurred after removal of the catheter on the first day, the catheter was reinserted for another day.

Women were asked to complete a questionnaire before surgery, and at 3–6 months and 12 months after treatment. All patients were asked to undergo a pelvic examination before treatment, at 6 weeks after treatment and when clinically indicated. Pelvic examination after 1 year was performed in women who were still followed up by their physician. Randomized patients were invited for examination after 12 months. During pelvic examinations, POP-Q stage was determined [15] in the lithotomy position and during a Valsalva manoeuvre. Other findings of the pelvic examination including vaginal erosion, vaginal discharge, atrophy, bleeding and urinary incontinence were also recorded.

The primary outcome of the study was the functional outcome evaluated using the Urogenital Distress Inventory (UDI) [16] after 12 months. The UDI is a questionnaire that has been validated in Dutch for evaluating prolapse-related symptoms. The UDI is a disease-specific questionnaire comprising 17 questions for assessing the presence and experienced discomfort of pelvic floor problems. The UDI consists of five domains: discomfort/pain, urinary incontinence, overactive bladder, genital prolapse, and obstructive micturition. The UDI scores were calculated for al five domains [16]. The questionnaire also includes versions of the Defecatory Distress Inventory (DDI) [17] and the Incontinence Impact Questionnaire (IIQ) [16]. The questionnaires also include questions about sexual function. If participants did not respond they were sent the questionnaire a second time. If they ignored both questionnaires, they were contacted by telephone to find the reason.

Additional interventions could include physiotherapy and incontinence surgery in the pessary group, and physiotherapy, incontinence surgery or surgery for recurrent prolapse in the surgery group. Long-term complications and side effects were recorded in both groups. The remaining study parameters included age, body mass index, parity, menopausal status, presence of incontinence and use of oestrogens. Urinary stress incontinence was diagnosed with the patient in the lithotomy position with a full bladder. The patient was asked to cough several times. If urinary leakage was seen, urinary incontinence was diagnosed. In patients with a cystocele, the cystocele was redressed and the examination was repeated.

### Sample size

Disease-specific quality of life as evaluated using the UDI questionnaire was the primary endpoint. A difference between the two interventions of ten points in the prolapse domain of the UDI 12 months after treatment was considered clinically relevant [18]. Assuming a standard deviation of the score in this domain of 15 points, 72 patients were needed to show a statistically significant difference in the primary outcome (power of 80%, α error 0.05) [18]. Taking into account a 10% attrition rate, 80 patients (40 in each arm) needed to be included. After the RCT was halted, we focused on the cohort group which was part of the trial, since randomization turned out to be very difficult. We included patients until there were at least 40 patients in both groups.

### Statistical analysis

The aim of the trial was to determine whether surgery was superior to pessary treatment in terms of the primary endpoint (prolapse domain of the UDI). For both nonrandomized and randomized groups, data were analysed according to the intention-to-treat principle. The domain scores were calculated for UDI, DDI and IIQ at baseline and after 12 months in both groups. The scores for these domains vary between zero and 100. A high score in a particular domain indicates more bothersome symptoms or worse quality of life. To examine differences between groups we used an unpaired *t* test or the Mann-Whitney test for continuous variables, depending on the distribution, and the chi-squared test was used for dichotomous variables. The Wilcoxon signed-ranks test was used to compare the domain scores before and after treatment in both groups separately. Two-sided significance tests were used, and *p* values <0.05 were considered to indicate statistical significance. For dichotomous outcomes, relative risks and 95% confidence intervals were calculated. All analyses were done with IBM SPSS statistics 22 (IBM, Armonk, NY).

## Results

**Fig. 1:**
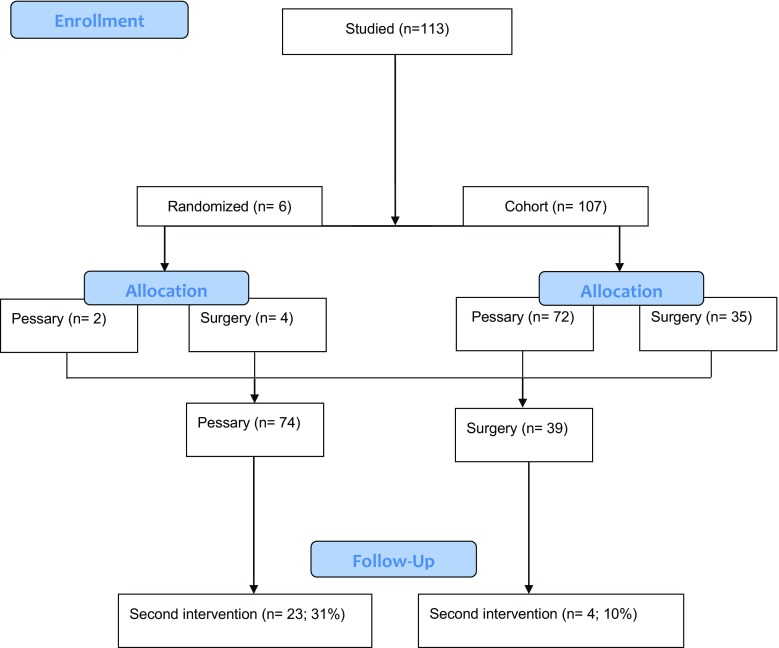
Patient flow chart

Between June 2009 and July 2014, 113 women were invited to participate in the study, of whom six gave informed consent for randomization, and 107 women expressed a treatment preference and participated in the prospective cohort study (72 preferred pessary treatment and 35 preferred prolapse surgery). The remaining six gave informed consent for randomization (two in the pessary group and four in the surgery group). As a result of the slow recruitment, we decided to halt the trial on 30 June 2014, leaving six women randomized and 107 women treated according to their preference. We performed an integrated analysis for randomized and nonrandomized patients, resulting in 74 women initially treated with pessary and 39 initially treated surgically (Fig. 1).

Table 1 shows the baseline characteristics of the study population. The pessary group was significantly older than the surgery group. Also, women in the pessary group had higher POP-Q stages of the anterior (*p* < 0.001) and posterior (*p* = 0.02) compartments than the surgery group. The surgery group did not include patients with POP-Q stage IV.

**Table 1:** Baseline characteristics of the study population

Characteristic		Group		*p-value*
		Pessary (*N* = 74)	Surgery (*N* = 39)	
Age (years), mean (range)		63.2 (60.4–65.9)	57.6 (53.8–61.4)	0.02
Body mass index (kg/m^2^), median (IQR)		25.8 (25.0–26.6)	24.6 (23.5–25.7)	0.07
Parity, *n*/*N* (%)				
0		0/74 (0)	0/39 (0)	0.84
1		9/74 (12)	4/39 (10)	
2		35/74 (47)	22/39 (56)	
3		19/74 (27)	8/39 (21)	
≥4		11/74 (15)	5/39 (13)	
Menopausal status, *n*/*N* (%)				
Premenopausal		4/67 (6)	6/35 (17)	0.07
Menopausal		0/67 (0)	1/35 (3)	
Postmenopausal		63/67 (94)	28/35 (80)	
Incontinence, *n*/*N* (%)				
No		33/70 (47)	17/38 (45)	0.07
Stress		24/70 (34)	20/38 (53)	
Urge		11/70 (16)	1/38 (3)	
Mixed		2/70 (3)	0/38 (0)	
Oestrogen use, *n*/*N* (%)				
Yes		5/50 (10)	2/37 (5)	0.45
No		45/50 (90)	35/37 (95)	
Preoperative POP-Q stage, %				
Anterior compartment	0	0	3	<0.01
	I	13	8	
	II	28	72	
	III	54	18	
	IV	6	0	
Apical compartment	0	1	0	0.34
	I	43	62	
	II	36	26	
	III	17	13	
	IV	3	0	
Posterior compartment	0	29	61	0.02
	I	39	18	
	II	25	16	
	III	3	5	
	IV	4	0	

Table 2 shows domain scores for the UDI, DDI, IIQ and sexual behaviour before treatment at baseline and after 12 months in both groups. The baseline overactive bladder scores were 11 (10th to 90th percentile 0–44) in the pessary group and 22 (10th to 90th percentile 0–58) in the surgery group (*p* = 0.02). The baseline pain/discomfort domain scores were 16 (10th to 90th percentile 0–63) in the pessary group and 33 (10th to 90th percentile 0–70) in the surgery group (*p* < 0.01). The baseline social impact of incontinence domain scores were 0 (10th to 90th percentile 0–22) in the pessary group and 11 (10th to 90th percentile 0–44) in the surgery group (*p* < 0.01).

**Table 2:** Disease-specific quality of life domain scores in both groups

	Baseline			12 months		
	Pessary group (*N* = 70)	Surgery group (*N* = 33)	*p* value	Pessary group (*N* = 60)	Surgery group (*N* = 26)	*p* value
Urogenital Distress Inventory^a^						
Overactive bladder	11.1 (0–44)	22.2 (0–58)	0.02	0.0 (0–33)	5.6 (0–56)	0.56
Incontinence	16.1 (0–44)	24.2 (0–73)	0.16	16.7 (0–35)	33.3 (0–50)	0.96
Obstructive micturition	0.0 (0–65)	16.7 (0–70)	0.02	0.0 (0–35)	0.0 (0–33)	0.39
Pain/discomfort	16.4 (0–63)	33.1 (0–70)	<0.01	0.0 (0–33)	0.0 (0–33)	0.74
Prolapse	33.3 (0–98)	33.3 (0–86)	0.64	0.0 (0–33)	0.0 (0–0)	<0.01
Recurrent bladder infections, *n* (%)						
Never	29 (41)	12 (36)	0.16	24 (40)	12 (46)	0.42
Once	4 (6)	7 (21)		2 (3)	3 (12)	
2 to 4 times	4 (6)	3 (9)		5 (8)	1 (4)	
> 4 times	1 (1)	0 (0)		1 (2)	0 (0)	
Defecatory Distress Inventory^a^						
Constipation	0.0 (0–23)	0.0 (0–47)	0.09	0.0 (0–17)	0.0 (0–55)	0.69
Obstructive defecation	0.0 (0–20)	8.3 (0–57)	0.28	0.0 (0–18)	0.0 (0–33)	0.21
Pain/discomfort	0.0 (0–33)	0.0 (0–17)	0.85	0.0 (0–33)	0.0 (0–22)	0.25
Incontinence	0.0 (0–17)	0 (0–17)	0.58	0.0 (0–33)	0.0 (0–0)	0.20
Incontinence flatus	0.0 (0–67)	33.3 (0–67)	0.09	0.0 (0–33)	33.3 (0–67)	0.18
Incontinence Impact Questionnaire^b^						
Physical	0.0 (0–48)	0.0 (0–50)	0.50	0.0 (0–33)	0.0 (0–13)	0.07
Mobility	11.1 (0–44)	16.7 (0–56)	0.26	0.0 (0–33)	0 (0–31)	0.71
Social	0.0 (0–22)	11.1 (0–44)	<0.01	0.0 (0–11)	0.0 (0–9)	0.86
Shame	0.0 (0–32)	0.0 (0–33)	0.23	0.0 (0–22)	0.0 (0–17)	0.99
Emotional	5.5 (0–43)	11.1 (0–67)	0.24	0.0 (0–37)	0.0 (0–11)	0.31
Sexuality, *n*/*N* (%)						
Sexual intercourse	42/64 (66)	25/32 (78)	0.21	35/53 (68)	21/27 (82)	0.21

The data presented are median scores (10th to 90th percentile) except as otherwise indicated

^a^ *0* not bothersome, *100* most bothersome

^b^ *0* best quality of life, *100* worst quality of life

The UDI prolapse domain score at 12 months was the primary outcome (Table 2). After 12 months, the median prolapse domain scores were 0 (10th to 90th percentile 0–33) in the pessary group and 0 (10th to 90th percentile 0–0) in the surgery group (*p* < 0.01), meaning that in the pessary group, 10% of the patients had a score of 33 or more in the domain “genital prolapse”, compared to all patients in the surgery group with a score of 0 in this domain. Other domain scores were not significantly different. The UDI prolapse domain scores had improved significantly in both groups at 12 months after treatment (*p* < 0.01).

Data concerning the initial treatment in the pessary group and the surgery group are presented in Tables 3 and 4, respectively. A ring pessary was used in most women in the pessary group (*N* = 64, 87%), followed by a shelf (Falk) pessary (*N* = 10, 14%). No other types of pessary were used during the follow-up period. There were no patients in the pessary group in whom we could not find a pessary that fitted at the first visit. Anterior colporrhaphy was the intervention performed in most women (74%) in the surgery group. Other techniques performed were laparoscopic hysteropexy (*N* = 1, 3%), sacrospinous fixation (*N* = 10, 26%), posterior colporrhaphy (*N* = 10, 26%), the Manchester-Fothergill procedure (*N* = 2, 5%), and transvaginal hysterectomy (*N* = 3, 8%).

**Table 3:** Data concerning the initial treatment in the pessary group

	Value
Type of pessary (*N* = 74), *n* (%)	
Falk	10 (14)
Ring	64 (87)
Pessary expulsion (*N* = 74), *n* (%)	10 (14)
Side effects (*N* = 74), *n* (%)	36 (49)
Vaginal discharge	15 (20)
Vaginal pain	10 (14)
Urinary incontinence	7 (9)
Erosion	3 (4)
Bleeding	1 (1)
Continuation rates (*N* = 74), *n* (%)	
4 weeks	60 (81)
3 months	60 (81)
6 months	47 (64)
1 year	44 (60)
Reason for discontinuation (*N* = 30), *n* (%)	
Pessary expulsion	7 (23)
Urinary incontinence	6 (20)
Vaginal pain	6 (20)
Vaginal discharge	5 (17)
No symptom reduction	5 (17)
Urinary retention	1 (3)

**Table 4:** Data concerning the initial treatment in the surgery group

	Value
Type of operation (*N* = 39), *n* (%)	
ACR	15 (39)
LH	1 (3)
SSF + ACR	9 (23)
SSF + ACR + PCR	1 (3)
ACR + PCR	7 (18)
MF + ACR	1 (3)
MF + ACR + PCR	1 (3)
TVH + ACR + PCR	1 (3)
TVH + ACR	1 (3)
TVH	2 (5)
Operative time (min), mean (95% CI)	64 (54–75)
Estimated blood loss (ml), median (IQR)	100 (100–300)
Hospital stay (days), median (IQR)	2 (2–3)
Complications during surgery (*N* = 39), *n* (%)	2 (5)
Bleeding, *n*	2
Complications during admission (*N* = 39), *n* (%)	13 (33)
Urinary tract infection, *n*	4
Bladder retention, *n*	8
Bleeding (reoperation), *n*	1

*ACR* Anterior colporrhaphy, *PCR* Posterior colporrhaphy, *LH* Laparoscopic hysteropexy, *TVH* Transvaginal hysterectomy, *MF* Manchester-Fothergill procedure, *SSF* Sacrospinous fixation

In the pessary group (Table 3), 49% of patients (36/74) had a side effect. The most common side effects were vaginal discharge (20%) and vaginal pain (14%). No urinary tract infections or vaginal ulcers were reported. During the follow-up period, 36% of patients (27/74switched to another type or size of pessary. Pessary expulsion occurred in 14% of patients (10/74). The continuation rate at 12 months was 60%. The reasons for discontinuation were expulsion, urinary incontinence, vaginal pain, discharge, urinary incontinence and no reduction in prolapse symptoms.

In the surgery group (Table 4), the mean operation time was 64 min (95% CI 54–75), and the median estimated blood loss was 100 ml (IQR 100–300 ml). The median hospital stay was 2 days (IQR 2–3 days). There were two complications recorded during surgery. In one woman, during anterior and posterior colporrhaphy diffuse bleeding occurred. This patient used vitamin K antagonists chronically which were bridged with subcutaneous low molecular weight heparin. The total estimated blood loss in this patient was 1,000 ml, but she recovered without needing transfusion. Another woman had a bleed directly after a sacrospinous fixation combined with an anterior colporrhaphy. She received four units of packed cells due to a bleeding near the sacrospinal ligament, which was successfully treated conservatively with vaginal tamponade. She developed a haematoma and was re-admitted to hospital for 1 day. The haematoma resolved spontaneously and was treated conservatively. Postoperative recovery was complicated by a urinary tract infection in four patients, by urinary retention in eight patients, and by a bleed in one patient who was re-admitted and re-operated upon 12 days after initial surgery because of an arterial bleed at the posterior vaginal wall.

A second intervention was performed in 31% of patients (23/74) in the pessary group, and in 10% of patients (4/39) in the surgery group (*p* = 0.01; Table 5. The additional interventions in the pessary group included POP surgery in 21 patients (28%), urinary incontinence surgery in 1 patient (1%) and physiotherapy in 1 patient (1%). The additional interventions in the surgery group included a pessary in 1 patient (3%), a pessary combined with physiotherapy in 2 patients (5%), and surgery for recurrent POP combined with physiotherapy in 1 patient (3%).

**Table 5:** Additional interventions

Intervention	Group		*p* value
	Pessary (*N* = 74)	Surgery (*N* = 39)	
Physiotherapy, *n*	1	0	
Pessary, *n*	0	1	
Prolapse surgery, *n*	21	0	
Incontinence surgery	1	0	
Total, *n* (%)	23 (31)	4 (10)	0.01
Combined			
Prolapse and incontinence surgery, *n*	0	0	
Prolapse surgery and physiotherapy, *n*	0	1	
Pessary and physiotherapy, *n*	0	2	
Time to second intervention (months), median (IQR)	3.0 (1.0–7.0)	10.0 (3.0–11.8)	0.17

## Discussion

### Main findings

In this study, we found that treatment preference limits patients’ willingness to undergo randomization. At 12 months the pessary group reported more symptoms in the prolapse domain of the UDI (the primary outcome) than the surgery group. Women in the pessary group were also more likely to need an additional intervention, and 28% needed surgical intervention in contrast to women in the surgery group of whom 5% needed reoperation. Younger patients, with a higher POP-Q stage and more severe urinary symptoms that affected their social life were more likely to choose POP surgery.

### Strengths and limitations

To our knowledge this is the first attempt to compare pessary and surgery as primary treatments for women with POP in a randomized trial. However, we were able to randomize only six women, since a large majority of the eligible women had a strong preference for one or other of the treatment options. Therefore, we decided to halt the trail and report the data as a prospective cohort study. Previous studies investigating women with POP have also shown strong patient preference for one or the other of two interventions [10]. Lamers et al. [10] found that the likelihood of preferring pessary treatment over surgery increases with increasing patient age. They concluded that surgery is preferred over pessary treatment as POP stage increases and if POP symptoms are more bothersome and affect general wellbeing, and that patients who are sexually active also tend to prefer surgery over conservative treatment [10]. In our study population, patient preference played an important role, to such a degree that randomization was very difficult.

Since most participating women were not randomized, selection bias was likely to have played a role. It is possible that doctors counselled women differently depending on patient characteristics such as age, comorbidity, sexual activity, POP stage and POP symptoms, and how they affect the woman’s daily activities. Because the groups were not allocated by randomization, the baseline characteristics were significantly different. The heterogeneity between the treatment groups is a reflection of normal daily practice. The pessary group in our study consisted of older women, as in previous studies [10]. The POP-Q stages of the pessary group were higher, in contrast to previous studies, but complaints were more bothersome in the surgery group, as has been found in previous studies [10].

The patients’ unwillingness to be randomized is an outcome in itself, as it reflects the strong differences between the two interventions with respect to invasiveness, risk profile and impact. The fact that the two treatment options are very different led to a strong preference for one or the other. However, in the OPUS trial [19], the randomization group and the patient-preference group did not show any differences in baseline characteristics or results. This might suggest that a RCT is not the only study design that can usefully add to the present literature, and the results of this prospective trial provide valuable conclusions. Following the advice of several epidemiologists, we did not perform separate analyses for our randomized and nonrandomized groups since the former was too small.

### Interpretation

This prospective cohort study comparing pessary treatment and surgery as primary treatments for POP generated some important lessons. As a randomized clinical trial comparing the two strategies turned out not to be feasible, our prospective study results can be used to counsel women. A strategy of pessary treatment followed by surgery if needed, or a strategy with immediate surgery both seem to be effective options for the treatment of POP in women.

Both groups reported very low scores for almost all domains of the UDI, DDI and IIQ, which suggests that both treatments are effective. The pessary group reported significantly more symptoms in the prolapse domain of the UDI. However, the median scores were still very low (0, 10th to 90th percentile 0–33, in the pessary group; 0, 10th to 90th percentile 0–0, in the surgery group), which is probably clinically irrelevant. Previous studies have also shown that pessaries are effective in improving pelvic floor dysfunction [6–9]. Most studies have shown improvements in both bulge and irritative bladder symptoms following pessary treatment [10], but two studies have also shown de novo stress urinary incontinence [9, 20]. Patient satisfaction rates with medium-term pessary use are high (70–92%) [20, 21]. Two prospective cohort studies comparing pessary treatment and surgery have shown similar improvements in urinary and bowel symptoms, sexual function, and quality of life at 12 months [11–14].

The continuation rate of pessary treatment after 12 months was 60%, whereas 72% of the women in the pessary did not have an indication for surgical intervention. This result is very important since it illustrates that both treatments are very effective in treating urinary, defaecation and prolapse symptoms, since the domain scores for the UDI, DDI and IIQ after 12 months were very low, with a median score of 0.0 for most domains. Prospective trials have shown continuation rates between 50% and 80% after 1 year and between 14% and 48% after 5 years [10]. However, evidence concerning reinterventions is lacking. Although no cost analysis was performed, these results suggests that pessary treatment is more cost effective, since less surgery was performed and the domain scores for the UDI, DDI and IIQ at 12 months were low in both groups.

The patient population was only 113 patients despite the long study period. As the study was non-funded, we were not able to register all patients who declined to participate. Therefore, we cannot provide reliable data about a denominator. We included patients until there were 40 patients in each group. Since there were only six randomized patients, we decided, following advice from several epidemiologists, not to perform a separate analysis for the two groups, but to analyse both groups together and present the data as a prospective cohort study instead of a RCT with a prospective cohort alongside. Nevertheless, the results of the study provide evidence on continuation rates, additional interventions, quality of life and patient preference. These outcomes will help individualize counselling on treatment options to provide better guidance to patients with symptomatic POP in the treatment decision process. Younger patients with a lower POP-Q prolapse stage but more urinary symptoms that affect their social life are more likely to choose POP surgery. This agrees with the results of previous studies. A review on the topic shows that the probability of choosing pessary treatment over surgery increases with increasing patient age in accordance with the results of this study. Surgery was preferred over pessary treatmen if POP symptoms are more bothersome and affect the general wellbeing of the patient [10].

### Conclusion

Women with POP stage II or higher treated with a pessary are bothered more by prolapse symptoms and more often undergo surgery in the first year of follow-up than patients who are treated surgically. However, pessary treatment allowed surgery to be avoided in 72% of women, although prolapse symptoms were less severe in those who had undergone surgery. These outcomes will help individualize counselling on treatment options and allow better guidance to patients with symptomatic POP in the treatment decision process.
